# Pathogenic fungi infection attributes of malarial vectors *Anopheles maculipennis* and *Anopheles superpictus* in central Iran

**DOI:** 10.1186/s12936-021-03927-4

**Published:** 2021-10-09

**Authors:** Seyed Hassan Moosa-Kazemi, Tahereh Sadat Asgarian, Mohammad Mehdi Sedaghat, Saeedeh Javar

**Affiliations:** 1 Department of Medical Entomology & Vector Control, School of Public Health, Tehran University of Medical Sciences, P.O. Box 6446-14155, Tehran, Iran; 2 Iranian Research Institute of Plant Protection, Agricultural Research, Education and Extention Organization (AREEO), Tehran, Iran

**Keywords:** Mosquito, Biocontrol agent, Entomopathogenic fungi

## Abstract

**Background:**

Due to the effect of synthetic and commercial insecticides on non-target organisms and the resistance of mosquitoes, non-chemical and environmentally friendly methods have become prevalent in recent years. The present study was to isolate entomopathogenic fungi with toxic effects on mosquitoes in natural larval habitats.

**Methods:**

Larvae of mosquitoes were collected from Central, Qamsar, Niasar, and Barzok Districts in Kashan County, Central Iran by standard dipping method, from April to late December 2019. Dead larvae, live larvae showing signs of infection, and larvae and pupae with a white coating of fungal mycelium on the outer surface of their bodies were isolated from the rest of the larvae and sterilized with 10% sodium hypochlorite for 2 min, then washed twice with distilled water and transferred to potato-dextrose-agar (PDA) and water-agar (WA) media and incubated at 25 ± 2 °C for 3–4 days. Larvae and fungi were identified morphologically based on identification keys.

**Results:**

A total of 9789 larvae were collected from urban and rural areas in Kashan County. Thirteen species were identified which were recognized to belong to three genera, including *Anopheles* (7.89%), *Culiseta* (17.42%) and *Culex* (74.69%). A total of 105 larvae, including *Anopheles superpictus *sensu lato (*s.l*), *Anopheles maculipennis s.l*., *Culex deserticola*, *Culex perexiguus*, and *Culiseta longiareolata* were found to be infected by *Nattrassia mangiferae*, *Aspergillus niger*, *Aspergillus fumigatus*, *Trichoderma* spp., and *Penicillium* spp. Of these, *Penicillium* spp. was the most abundant fungus isolated and identified from the larval habitats, while *An*. *superpictus s.l*. was the most infected mosquito species.

**Conclusions:**

Based on the observations and results obtained of the study, isolated fungi had the potential efficacy for pathogenicity on mosquito larvae. It is suggested that their effects on mosquito larvae should be investigated in the laboratory. The most important point, however, is the proper way of exploiting these biocontrol agents to maximize their effect on reducing the population of vector mosquito larvae without any negative effect on non-target organisms.

## Background

Transmission of malaria, filariasis, Japanese encephalitis, dengue fever, and other arboviral diseases by mosquitoes has turned mosquitoes into the most important group of arthropods in medicine and health [1]. In Iran, mosquitoes are vectors of two protozoan, two bacterial, four filarial, and seven arboviral diseases [2, 3]. There are 70 species and eight (or 12) genera of Iranian mosquitoes depending on the classification of the tribe Aedini [4]. *Anopheles* species are responsible for the transmission of malaria, but the majority of mosquito species from the genera of *Culex* and *Aedes* are responsible for the transmission of arboviruses to humans [5, 6].

Globally, in 2019, there were an estimated 229 million malaria cases in 87 malaria-endemic countries. The disease is a major endemic infectious disease in Iran, especially in the south and southeastern provinces, including the Sistan-Baluchistan, Hormozgan and Kerman Provinces [7–12].

*Anopheles* species are responsible for the transmission of malaria. So far, seven malaria vectors have been recognized and reported in Iran, including *Anopheles stephensi*, *Anopheles culicifacies*, *Anopheles dthali*, *Anopheles fluviatilis*, *Anopheles superpictus*, *Anopheles maculipennis*, and *Anopheles sacharovi* [13]. The first five of these vectors can be found in the southeast of the country, together with the majority of malaria cases. Also, *Anopheles pulcherrimus* has been considered as a potential malaria vector in this area based on immunological parasite detection [two-site immunoradiometric assay (IRMA)] [14].

*Anopheles maculipennis *sensu lato (*s.l*.) is distributed in Eurasia and North America and comprises nine Palearctic members [15, 16]. Some research indicated the occurrence of this malaria vector in Central Iran, the Caspian coast in the north, and North-West Iran [17–19].

*Anopheles superpictus s.l.* is distributed in Europe, Asia and North Africa [20–24]. This species is one of the seven species of malaria vectors and reported in the Iranian Plateau, the slopes of the Alborz Mountains and southern Zagros, as well as the coastal plains of the Caspian Sea and the Persian Gulf in both malaria-endemic and non-endemic areas [8, 21]. Oshaghi et al*.* in 2008 reported three genotypes X, Y and Z in Iran. Interestingly, while the sympatric Y and Z genotypes appear to be exclusive to the populations from the southeastern part of the country, genotype X is geographically separated, and present in the North, the West, the South and the Central territories [22].

One of the goals of control methods is to reduce the size of vector populations. There is a risk of insecticide resistance and off-target effects on other arthropod species in chemical control [25]. Biological control is biodegradable and ecologically friendly [26]. Entomopathogenic fungi were first used on *Anopheles gambiae* with a fungus from the genus of *Coelomomyces* [27]. Weiser et al*.* reported *Coelomomyces irani* from *An. maculipennis* in Iran [28]. Azari-Hamidian and Abaei reported *Coelomomyces* sp. from the larvae of *An. culicifacies s.l.* in Sistan and Baluchistan Province, southeast Iran, where 5.8% of larvae were infected with the fungus [29].

The use of pathogenic insect fungi against mosquito larvae has been reported in many studies, and fungi are proven an effective way of killing mosquito larvae [30–34]. The use of *Beauveria bassiana* for control of *Aedes aegypti* [31] and *Lagenidium giganteum* in California targeted to control *Culex tarsalis* [32] reduced the survival rate, blood-feeding, fecundity, and disease transmission power of targeted mosquitoes. Some insect pathogenic fungi have been used effectively in the laboratory, small-, and large-scale field studies to control vector mosquitoes especially in *Culex* [35, 36], *Mansonia* [37], and *Anopheles* species [32] and have a wide range of species diversity. This group of pathogens is found among all phyla of fungi. The Ascomycota is the largest group of fungi. This group is extremely ecologically diverse, just like the pathogenesis pathogen of plants, animals and humans. Pathogenic insect ascomycetes include a large group of fungi that attack a wide range of insects and are the most common insect pathogens [38]. The entomopathogenic ascomycete fungi, including *Metarhizium anisopliae* and *Beauveria bassiana* have been reported as insecticides [39]. In many studies, spores and secondary metabolites of insect pathogenic fungi have been reported as biocontrol agents against mosquitoes [34, 40–42]. The fungal hyphae produce endotoxins and penetrate through the larval body. These toxins cause larval damage and toxicity in the haemocoel and larval mosquito guts [43]. Metabolites of *Beauveria bassiana* caused changes in the body and tissues of treated *Culex pipiens* larvae, especially in the cuticle and midgut [44].

The present study was to isolate and identify entomopathogenic fungi associated with mosquito larvae in Kashan County, Central Iran, and their infection and effects on mosquito larvae.

## Methods

### Study area

Kashan County is located in central Iran, north of Isfahan Province. This county has four districts, including Central (51°24′43.2″ E, 34°00′16.0″ N), Qamsar (51°27′45.8″ E, 33°45′30.5″ N), Niasar (51°08′47.6″ E, 33°58′39.3″ N), and Barzok (51°13′44″ E, 33°47′32″ N) Districts. The climate of the county varies depending on ups and downs. The uplands are cold, foothills are temperate, and the plains, especially on the margins of the desert, are tropical [45].

**Fig. 1:**
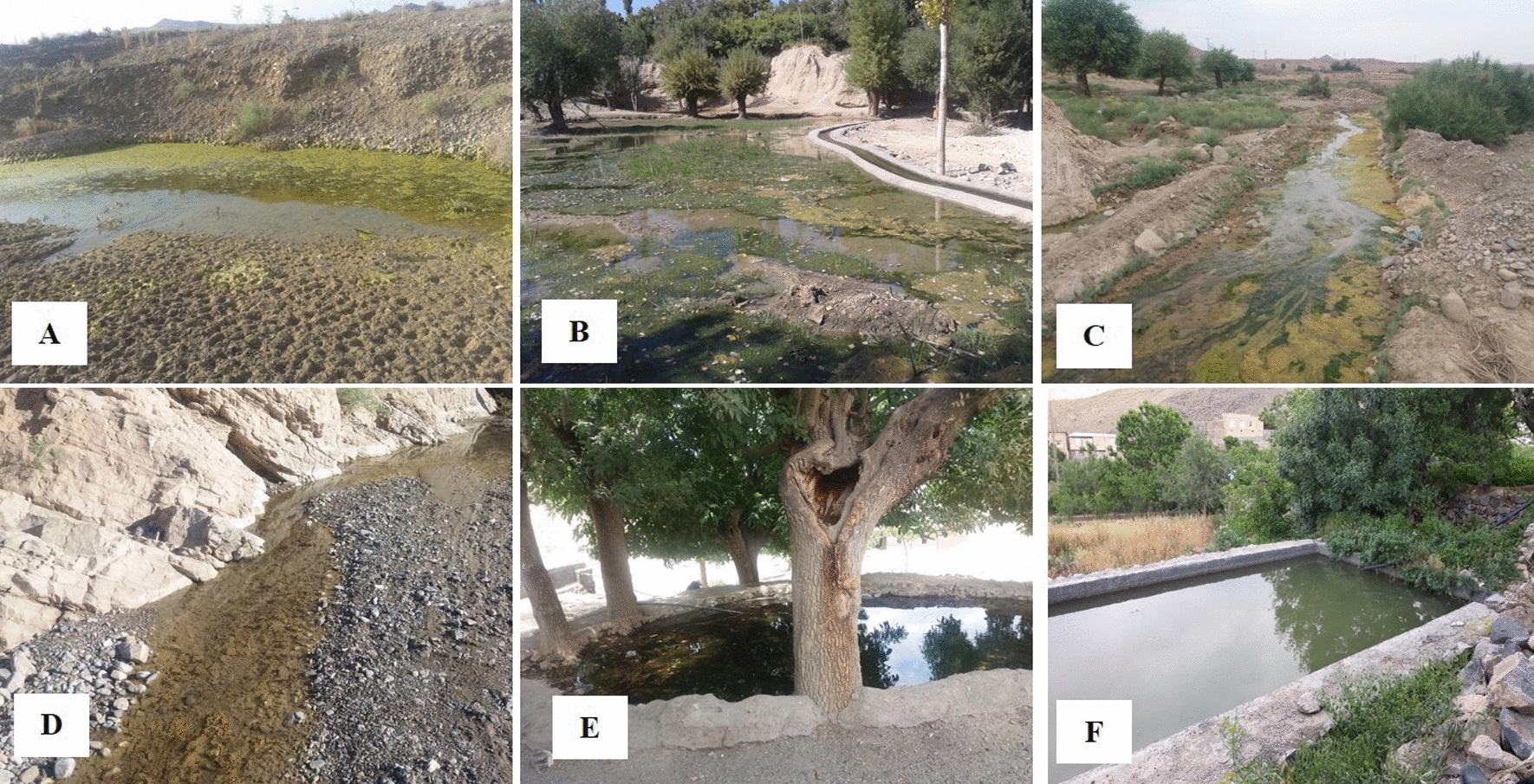
General views of mosquito larval habitats in Kashan County, central Iran, 2019

A total of 23 larval habitats were selected in Central, Qamsar, Niasar, and Barzok Districts. These larval habitats are natural or artificial, permanent or temporary, with or without vegetation, sunlight or shaded, and clear or stagnant water (Fig. 1).

### Larval sampling

Using a standard 350-ml capacity mosquito dipper, larvae and pupae of the mosquitoes were collected from April to late December 2019. Twenty dips were taken in each larval habitat in the morning (08:00–12:00 h) or afternoon (15:00–18:00 h). For sampling larvae from small water bodies, an eyedropper was used. The collected larvae and pupae were transferred into clean plastic jars along with larval habitat water, stored in a cool box with temperature ranging from 8 to 15 °C, the date, collection site and habitat type of larvae recorded with special code on the containers and relevant forms, and then transferred to the medical entomology laboratory of Tehran University of Medical Sciences (TUMS) in less than 5 h.

Immediately after transferring larvae to the laboratory, larvae were observed under a stereomicroscope. Dead larvae, live larvae showing signs of infection, and larvae and pupae with a white coating of fungal mycelium on the outer surface of their bodies were isolated from the rest of the larvae and maintained at 4 °C for isolation and diagnosis of fungi associated with mosquito larvae. Other mosquito larvae were transparent in lactophenol, individually mounted in Berlese's fluid on a microscope slide and identified based on a Culicidae identification key of Iran at the species level [46].

### Isolation and diagnosis of fungi associated with mosquito larvae

**Fig. 2:**
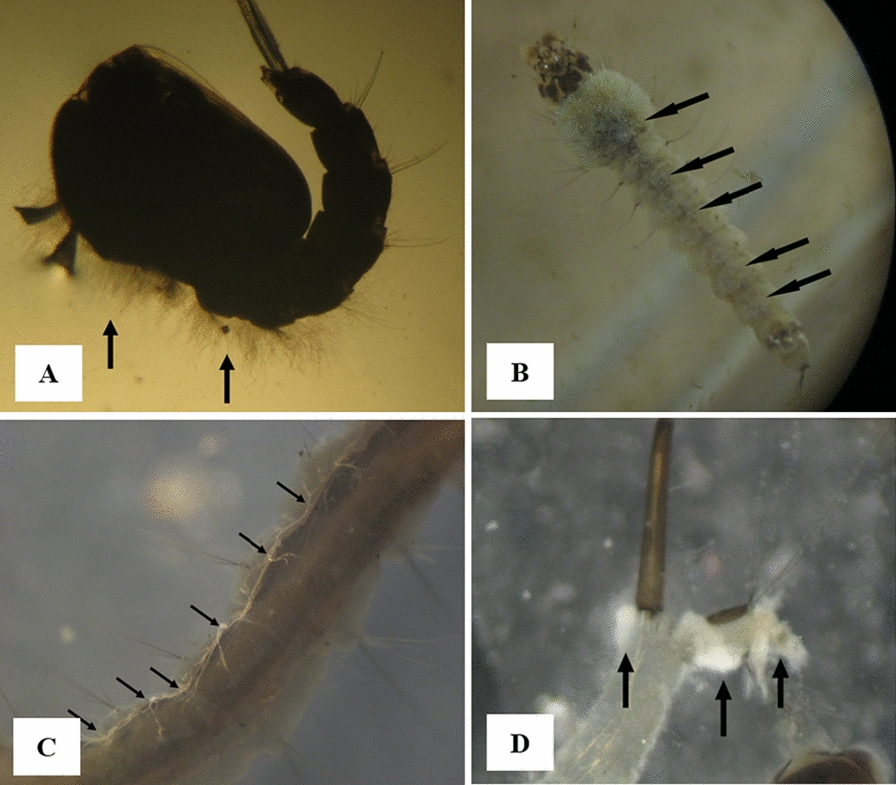
Stereomicroscopic view of pathogenic fungi on the 4th instar larvae and pupae of mosquitoes in Kashan County, central Iran, 2019. Fungal mycelium on **A** *An. superpictus s.l.* pupa*,* **B** *An. superpictus s.l.* larva*,* **C** *Culiseta longiareolata* larva **D** *Culex* spp. larva. (Arrows show fungal hyphae)

**Fig. 3:**
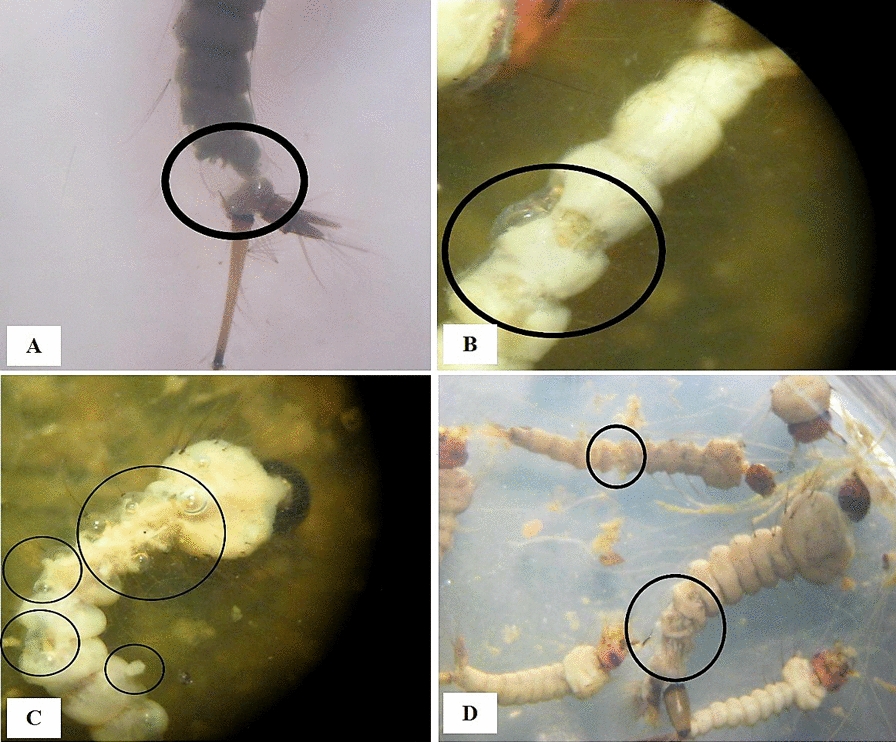
**A**–**D** Stereomicroscopic view of 4th instar mosquito larvae, Kashan County, central Iran, 2019. The circles indicate crumbled epithelial layer of the outer cuticle, shrinkage of the larvae, and bumps on the surface of the larval bodies

A white coating of fungal mycelium was observed on the surface of some of the larvae and pupae (Fig. 2). These larvae and pupae were removed from the water of the larval habitat at the laboratory and sterilized with 10% sodium hypochlorite for 2 min (to remove surface contaminants that conflict with the main pathogen), then washed twice with distilled water; the remaining water was removed and passed through the filter paper sterilizer [47]. They were then transferred to potato-dextrose-agar (PDA) and water-agar (WA) media. Parts of the body of some other larvae were degraded or broken, or the outer epithelial layer of the larvae were cut and collapsed. Consequently, the outer surface of the larvae was wrinkled (Fig. 3). To determine the possibility of fungal infection, they were transferred to PDA and WA media after surface disinfection, then incubated the Petri dishes in the incubator at 25 ± 2 °C for 3–4 days. Fungi were identified based on phenotypic characteristics and characteristics of the culture medium, such as shape and colour of the fungus colony, filament growth pattern, as well as microscopic properties such as shape, size and colour of spores, mycelium and conidiophore structure [48].

## Results

### Larval sampling results

A total of 9789 larvae were collected from urban and rural areas of Central, Qamsar, Niasar, and Barzok Districts in Kashan County. Three genera: *Anopheles* (7.89%), *Culiseta* (17.42%), and *Culex* (74.69%), consisting of 13 species were identified (Table 1). Some mosquito specimens were deposited in the Museum of Medical Entomology, TUMS.

**Table 1:** Mosquito species collected in Kashan County, central Iran, 2019

Districts	Species
Central	*An. superpictus s.l.* (159), *Cs. longiareolata* (566), *Cx. theileri* (1028), *Cx. deserticola* (175), *Cx. hortensis* (150), *Cx. mimeticus* (15), *Cx. perexiguus* (205), *Cx. pipiens* (2651)
Qamsar	*An. maculipennis s.l.* (54), *An. superpictus s.l.* (453), *Cs. longiareolata* (355), *Cx. pipiens* (733), *Cx. theileri* (1233), *Cx. deserticola* (105), *Cx. hortensis* (31), *Cx. mimeticus* (39), *Cx. perexiguus* (29)
Niasar	*An. maculipennis s.l.* (15), *An. claviger* (1), *Cs. annulata* (43), *Cs. subochrea* (2), *Cs. longiareolata* (460), *Cx. deserticola* (68), *Cx. hortensis* (40), *Cx. pipiens* (37), *Cx. theileri* (176), *Cx. perexiguus* (71)
Barzok	*An. maculipennis s.l.* (22), *An. superpictus s.l.* (57), *An. turkhudi* (1), *Cs. longiareolata* (280), *Cx. deserticola* (92), *Cx. hortensis* (33), *Cx. pipiens* (237), *Cx. mimeticus* (14), *Cx. theileri* (118), *Cx. perexiguus* (31)

### Fungi associated with mosquito larvae

Five species of fungi were isolated from mosquito larvae (Table 2). These fungi were isolated from larvae and pupae obtained from natural larval habitats in Qamsar and Barzok Districts. Five out of 13 mosquito species were found to be infected by fungi. A total of 105 larvae revealed morphological or behavioural manifestations of infection and fungal mycelia were detected in all of these larvae.

**Table 2:** Number and percentage of live and dead mosquito larvae and pupae infected with fungi collected from Kashan County, central Iran, 2019

Districts	Fungi isolated	Number and percentage of mosquito larvae infected						Number and percentage of mosquito pupae infected			
		Live					Dead	Live			Dead
Qamsar	*Nattrassia mangiferae*	*An. superpictus s.l.* (4) (0.88%)					0	*An. superpictus* s.l*.* (1) (0.22%)			0
	*Aspergillus niger*	0					*An. maculipennis s.l.* (2) (3.70%), *An. superpictus s.l.* (12) (2.65%)	0			0
	*Aspergillus fumigatus*	*An. superpictus s.l.* (2) (0.44%)				*An. superpictus s.l.* (5) (1.10%), *An. maculipennis s.l.* (1) (1.85%),		0		0	
Barzok	*Aspergillus niger*	0			*An. maculipennis s.l.* (2) (9.1%), *An. superpictus s.l.* (4) (7.02%), *Cx. deserticola* (3) (3.26%), *Cx. perexiguus* (3) (9.68%)			0	0		
	*Aspergillus fumigatus*	*An. maculipennis s.l.* (1) (4.55%), *An. superpictus s.l.* (4) (7.02%)		0				0	0		
	*Penicillium* spp.	*An. maculipennis s.l.* (3) (13.64%), *An. superpictus s.l.* (25) (43.86%), *Cs. longiareolata* (14) (5%), *Cx. deserticola* (9) (9.78%), *Cx. perexiguus* (6) (19.35%)	0					0	0		
	*Trichoderma* spp.	*An. superpictus s.l.* (2) (3.51%), *Cs. longiareolata* (2) (0.71%)	0					0	0		

**Fig. 4:**
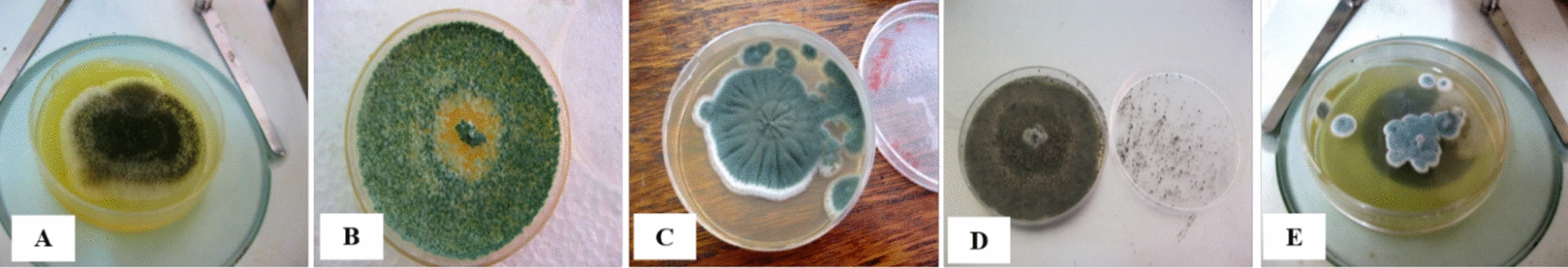
Fungi isolated from mosquito larvae and grown in PDA, Kashan County, central Iran, 2019. **A** *Aspergillus niger,* **B** *Trichoderma* spp., **C** *Aspergillus fumigatus*, **D** *Nattrassia magniferae,* **E** *Penicillium* spp.

**Fig. 5:**
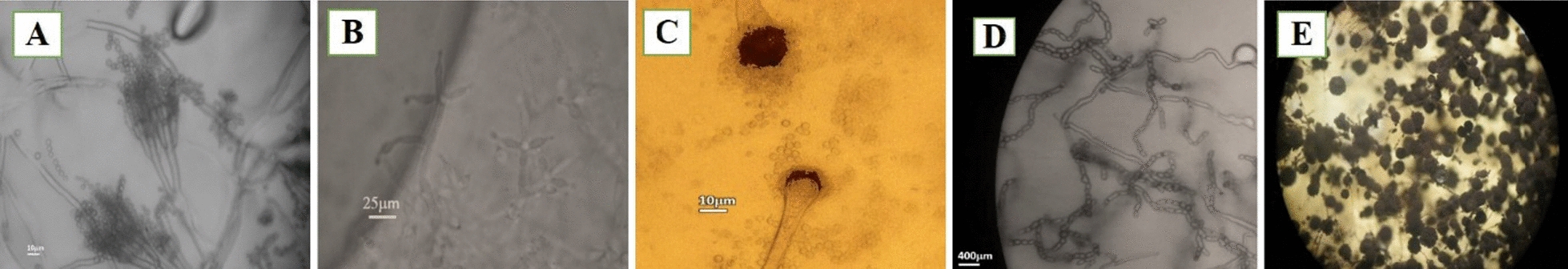
Microscopic image of fungi medium isolated from mosquito larvae in Kashan County, central Iran, 2019. **A** *Penicillium* spp., **B** *Trichoderma* spp., **C** *Aspergillus fumigatus*, **D** *Nattrassia magniferae* and stereomicroscopic view of **E** *Aspergillus niger*

**Fig. 6:**
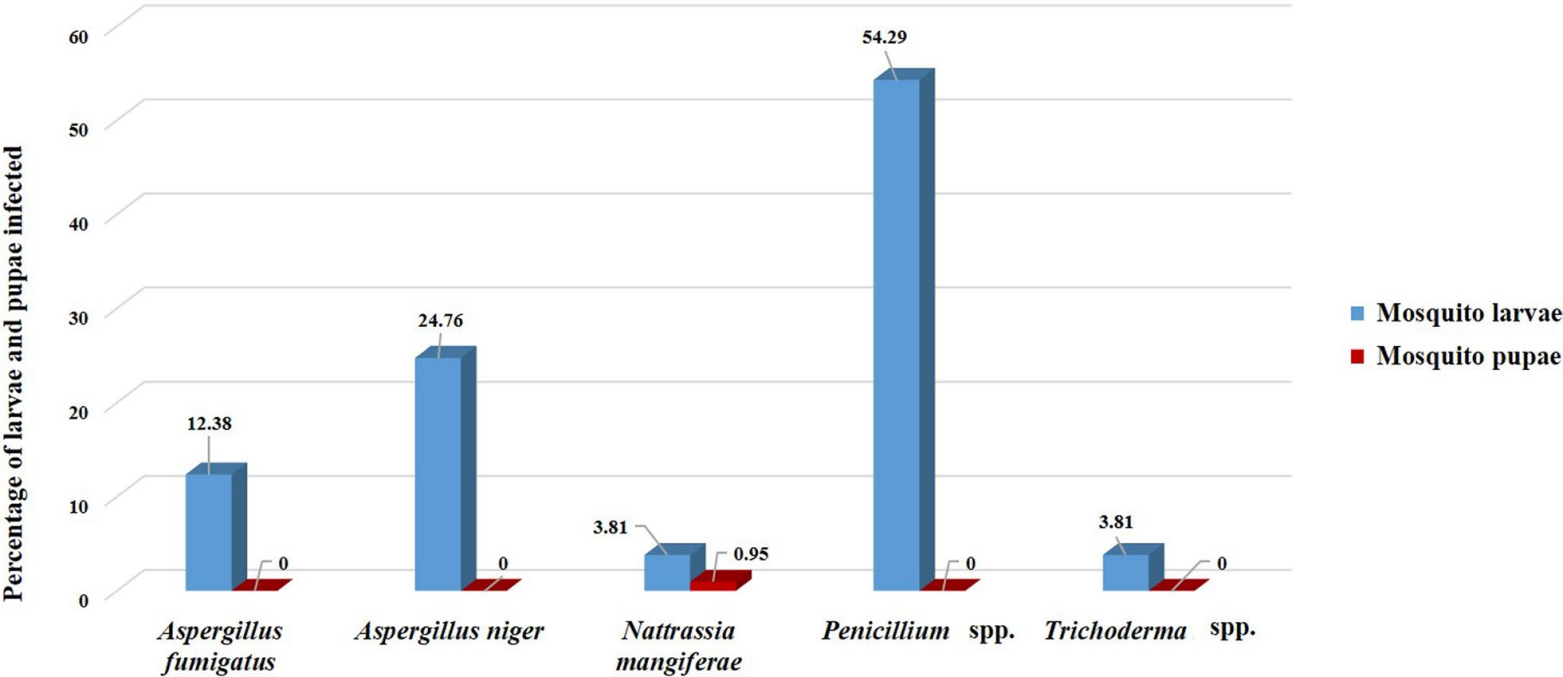
Percentage of fungi detected from infected larvae and pupae in Kashan County, central Iran, 2019

*Nattrassia mangiferae* was isolated only from *An. superpictus* s. l*.* larvae or pupae in Qamsar District in August. The white hyphae of *Aspergillus niger*, *Aspergillus fumigatus* and *Trichoderma* spp. had grown on the surface of the larvae, and penetrated the body. *Penicillium* spp. was identified from larvae whose parts of their bodies were wrinkled, degenerated or broken (Figs. 4, 5). In this study, *Penicillium* spp. was isolated from 57 mosquito larvae (54.29% of infected larvae) and it was the most abundant fungus isolated, and identified from larval mosquito habitats in Kashan County (Fig. 6). This fungus was identified from larvae collected from a natural larval habitat with vegetation in Barzok. *Anopheles superpictus* s. l. had the highest number of larvae infected with the fungi in all larval habitats, and from 105 infected larvae collected, 59 larvae were related to this species (Table 2).

## Discussion

Insect pathogenic fungi can grow in liquid and solid environments, and their spores can attack and kill mosquito larvae [49]. In the present study, five fungi species were identified from mosquito pupae or larvae. All of these fungi were isolated from larvae and pupae collected from natural larval habitats.

### Effects of fungi mycelia and secondary metabolites on mosquito larvae

*Nattrassia mangiferae* is common in the tropics and is best known as a plant pathogen (the cause of die‐back and trunk cankers in trees). It can also cause fungal infections in human nails [50]. This fungus had not previously been isolated from insects and this is the first report from mosquito larvae and pupae.

*Aspergillus* has more than 180 species, some of them are pathogenic or allergenic to humans and animals. In different studies, several species of this fungus have been reported in mosquito larvae. Turky et al. [47] isolated *Aspergillus candids*, *Aspergillus niger*, and *Aspergillus terreus* from *Culex quinquefasciatus* larvae. Balumahendhiran et al. [51] examined secondary metabolites of *Aspergillus flavus* and *Aspergillus fumigatus* in the control of *Aedes aegypti*, *An. stephensi* and *Cx. quinquefasciatus* larvae. Species of *Aspergillus* produce a variety of secondary metabolites, including aflatoxins. Secondary metabolites of *Aspergillus fumigatus* had the highest toxicity to *Cx. quinquefasciatus* and *An. stephensi* larvae. Secondary metabolites of *Aspergillus niger* were also effective against the larvae of these three mosquito species [30]. In this study, it was also found that two species of *Aspergillus*, including *Aspergillus niger* and *Aspergillus fumigatus*, can grow in an aquatic environment on mosquito larvae and infect them.

In the present study, *Trichoderma* spp. were also isolated and identified from mosquito larvae. This fungus is capable of attacking other organisms and microorganisms by producing antibiotics and other extracellular enzymes. Because of this ability, *Trichoderma* spp. has been known as biocontrol agent of plant pathogens for about 70 years [52]. This fungus is widely used in agriculture to control plant diseases as well as to increase crop yield. Podder and Ghosh investigated the effect of *Trichoderma asperellum* against anophelinae larvae and their study was the first report the use of *Trichoderma asperellum* as mosquito larvicides. They observed that the internal tissues of the larvae were destroyed after larval death [53]. It has been confirmed that some fungal toxins can cause tissue damage and dehydration of the host tissues [54].

In a study in 2016, researchers reported *Metarhizium brunneum* blastospores kill *Aedes* larvae much faster than conidia of this fungus in natural habitat, in freshwater. Blastopores easily penetrated the larval cuticle and resulted in rapid larval death. Conidia cause stress-induced mortality, which takes a slightly longer time [49].

### Effects of fungi on morphological or behavioural manifestations of mosquito larvae

In the present study, *Penicillium* spp. was isolated from wrinkled and degenerated larvae and the larvae whose parts of their cuticle were destroyed. Ragavendran et al. [55] studied the effect of larvicidal of seven fungal isolates and their metabolites on *Ae. aegypti* and *Cx. quinquefasciatus *in vitro and reported that *Penicillium* spp. had the best larvicidal effect compared to other fungi. The mycelia extract of this fungus had toxic effects on many parts of the larval body, including thorax, abdomen, anal gills, such as the loss of external hair, crumbled epithelial layer of the outer cuticle, and shrinkage of the larvae. After 30 min of exposure of the larvae to the fungal metabolites, the behavioural symptoms of the treated larvae were observed, including upward, downward, horizontal, and vertical movements of the larvae and damage at the bottom of the larval body. Damage to the cuticle layers was also one of the morphological changes in the treated larvae. In this study also, *Penicillium* spp. was isolated from larvae that had morphological manifestations in the cuticle layers. Lethargy and inactivity were among behavioural manifestations observed in these larvae.

Infection with all fungi identified in this study was present in *An. superpictus s.l.* larvae. Omrani et al. reported the first case of a microsporidium infection (a microsporidium species from the genus *Parathelohania*) in *An. superpictus s.l.* from Iran [56]. *Parathelohania legeri* was reported in *An. maculipennis s.l*. about 110 years ago [57].

In addition to parasitic effects and their potential for mosquito control, mosquito-associated fungi also have non-pathogenic interactions with mosquitoes, such as the impact on breeding site selection and impact on larval and adult feeding behaviour. It has been demonstrated that secondary metabolites produced by *Trichoderma viride* have effects on attracting gravid *Cx. quinquefasciatus* females and find oviposition sites [58, 59].

There are several reports of insecticide resistance in malaria and West Nile vectors in Iran [13, 60–75]. Studies on non-pathogenic fungi of mosquitoes are very scarce and have not been done in Iran. A study of the impact of pathogenic and non-pathogenic fungi on the behaviour of mosquitoes can help to develop new vector control strategies. In addition, some fungi are recommended for combinations of insecticides for indoor residual spraying as adult control. Using these fungi in combination with other vector control measures is appropriate for decision-makers [76–78].

## Conclusion

This study did not examine the lethal effects of these fungi on larvae in the laboratory, and reports only natural fungi infection in mosquito larvae in their natural habitats. Therefore, it is suggested that their effects on mosquito larvae be investigated in the laboratory. The most important point, however, is the proper way of exploiting these biocontrol agents to maximize their effect on reducing the population of vector mosquito larvae without any negative effect on non-target organisms.

## Data Availability

Not applicable.

## Abbreviations

PDA: Potato-dextrose-agar
WA: Water-agar
